# Children with speech sound disorder: comparing a non-linguistic auditory approach with a phonological intervention approach to improve phonological skills

**DOI:** 10.3389/fpsyg.2015.00064

**Published:** 2015-02-04

**Authors:** Cristina F. B. Murphy, Luciana O. Pagan-Neves, Haydée F. Wertzner, Eliane Schochat

**Affiliations:** Department of Physical Therapy, Speech-Language Pathology and Occupational Therapy, Center for Teaching and Research, School of Medicine, University of São PauloSão Paulo, Brazil

**Keywords:** speech sound disorder, phonology impairment, language therapy, auditory stimulation, children

## Abstract

This study aimed to compare the effects of a non-linguistic auditory intervention approach with a phonological intervention approach on the phonological skills of children with speech sound disorder (SSD). A total of 17 children, aged 7–12 years, with SSD were randomly allocated to either the non-linguistic auditory temporal intervention group (*n* = 10, average age 7.7 ± 1.2) or phonological intervention group (*n* = 7, average age 8.6 ± 1.2). The intervention outcomes included auditory-sensory measures (auditory temporal processing skills) and cognitive measures (attention, short-term memory, speech production, and phonological awareness skills). The auditory approach focused on non-linguistic auditory training (e.g., backward masking and frequency discrimination), whereas the phonological approach focused on speech sound training (e.g., phonological organization and awareness). Both interventions consisted of 12 45-min sessions delivered twice per week, for a total of 9 h. Intra-group analysis demonstrated that the auditory intervention group showed significant gains in both auditory and cognitive measures, whereas no significant gain was observed in the phonological intervention group. No significant improvement on phonological skills was observed in any of the groups. Inter-group analysis demonstrated significant differences between the improvement following training for both groups, with a more pronounced gain for the non-linguistic auditory temporal intervention in one of the visual attention measures and both auditory measures. Therefore, both analyses suggest that although the non-linguistic auditory intervention approach appeared to be the most effective intervention approach, it was not sufficient to promote the enhancement of phonological skills.

## INTRODUCTION

Speech sound disorder (SSD) is defined as a developmental disorder characterized by articulatory and/or phonological difficulties that affect a child’s ability to be understood by others, leading to reduced speech intelligibility, in the absence of other cognitive, sensory, motor, structural, or affective issues (Shriberg, 2003; Raitano et al., 2004; McGrath et al., 2007). It is currently well-established that, in most cases, the primary characteristics of SSD are difficulties in acquiring the phonological representations of speech sound systems in addition to deficits in speech perception and phonological tasks (Bird and Bishop, 1992; Leitao and Fletcher, 2004; Kenney et al., 2006; Fey, 2008). Despite the overlap of symptoms between SSD and language impairments, such as specific language impairment (SLI), SSD have their own characteristics (primarily increased substitution or omission of sounds from words compared to same-aged peers and speech production errors) and constitute the largest group of speech and language impairments observed in children (Shriberg and Kwiatkowski, 1982; Shriberg et al., 1994; Broomfield and Dodd, 2004; Tkach et al., 2011). According to Shriberg et al. (1999), the prevalence of SSD is ∼2–13%, and the rate of comorbidity between SSD and SLI in 6-years-old children, for instance, is 0.51%.

Several studies have investigated the effects of different intervention approaches on phonological impairments in children with SSD. For many years, the most common treatment approach in speech language pathology was the traditional articulation approach (Van Riper, 1939), which focuses on how to articulate individual phonemes to improve speech intelligibility. Over time, several phonological intervention approaches were incorporated in speech therapy by focusing on the phonological representations of speech sound systems, including phonemic awareness, vocabulary, and/or phonological memory tasks. Williams et al. (2010) documented 23 different intervention approaches for children with SSD, with the cycles approach (Hodson and Paden, 1983, 1991) and the core vocabulary approach (Holm et al., 2005) as examples of recognized phonological therapies. The Cycles Phonological Remediation Approach (Hodson and Paden, 1983, 1991) aims to increase a child’s intelligibility by facilitating the emergence of the following primary target patterns for beginning cycles such as final consonants, clusters, velars, and liquids. The Core Vocabulary approach establishes consistency of production and enhances consonant and vowel accuracy. According to Crosbie et al. (2006), this approach is effective for children with an inconsistent phonological disorder.

As previously mentioned, numerous studies have demonstrated that one symptom of SSD is speech perception deficits. However, the role of this deficit in developmental phonological disorders remains unclear. Since the 1980s, research has supported the hypothesis, initially proposed by Tallal and Piercy (1973), that an auditory-sensory deficit, more specifically, an auditory temporal processing deficit, may be the underlying cause of speech perception deficits (Tallal and Piercy, 1973; Tallal, 1980; Tallal et al., 1996; Fitch et al., 1997; Habib, 2000; Ingelghem et al., 2001; Share et al., 2002; Murphy and Schochat, 2009a,b). This auditory temporal processing difficulty can be described as a limited ability to process “acoustic elements of short duration” such as consonants with rapid formant transitions. Thus, children with language impairments, including SSD, would have difficulties perceiving and distinguishing these sounds properly within the speech spectrum and subsequently developing the phonological representation of each one to produce them properly. Based on this hypothesis, a large number of studies have investigated the effects of auditory temporal training on language and phonological skills (Merzenich et al., 1996; Tallal et al., 1996; Kujala et al., 2001; Hayes et al., 2003; Cohen et al., 2005; Russo et al., 2005; Strehlow et al., 2006; Gaab et al., 2007; Lakshminarayanan and Tallal, 2007; Gillam et al., 2008; Given et al., 2008; Murphy and Schochat, 2011; Heim et al., 2013). Despite this body of research, the extent to which auditory perceptual learning is generalized to higher phonological skills remains controversial and this controversy is often discussed in terms of methodology issues.

In the research conducted by Tallal et al. (1996), for instance, the trained group was composed of children with both speech and language impairments (described by the authors as language-learning impairments). Therefore, combining children with SSD and SLI together might confound the observation of a relationship between pure speech perception deficits and auditory temporal processing skills. In addition, there is no consensus as to whether the changes in language skills that follow auditory training are due to specific auditory-sensory learning or to a general enhancement in cognitive skills. Numerous studies have demonstrated that auditory training can also promote improvement in cognitive skills (especially with regard to working memory and attention) in addition to the enhancement of auditory-sensory skills (Mahncke et al., 2006; Adcock et al., 2009; Murphy et al., 2011).

Although a great number of studies have addressed the effectiveness of auditory and phonological intervention approaches on the language skills of children with either SLI or dyslexia, only a few studies have investigated the effect of these intervention approaches in the speech production and phonological awareness skills of children with SSD. Lousada et al. (2012) described the presence of learning generalization in a study evaluating the effectiveness of a phonological intervention approach and an articulation intervention approach in children with SSDs. Either a generalization probe of the trained sound or phonological process to five non-intervention words was used. The authors demonstrated that the children in the phonological group showed greater generalization to untreated words than those who received articulation therapy. No study has investigated the efficacy of the auditory training or even attempted a direct comparison of the effectiveness of auditory and phonological intervention approaches on speech production and phonological awareness skills. Baker and McLeod (2011) for example, mentioned that few studies have demonstrated that one intervention approach is more efficient to another with a specific disorder group. Besides, most of the studies reporting efficacy studies were quasi-experimental designs or no experimental, indicating the need of more controlled studies including groups of children and randomized controlled interventions (Brumbaugha and Smita, 2014).

Therefore, the aim of the present study is to compare the effect of an auditory and phonological intervention approach on speech production and phonological awareness skills in children with SSD. Taking into account previous studies demonstrating a strong link between impaired phonological processing and SSD as well as the hypothesis associating speech perception deficits to an auditory-sensory impairment, we will be able to explore the real contribution of phonological skills as well as the auditory-sensory aspects in language skills by comparing both intervention approaches. We also aim to investigate the extent to which both interventions may improve other deficits present in children with SSD, including sustained attention (Murphy et al., 2014) and phonological working memory deficits (Adams and Gathercole, 1995). We hypothesized that each of the interventions will improve the performance in the trained tasks (auditory and phonological skills) and result in learning transfer to associated tasks in the same or different domains (language, auditory, memory, and attention skills).

## MATERIALS AND METHODS

This study was conducted at the Department of Physical Therapy, Speech-Language Pathology and Occupational Therapy in the School of Medicine (FMUSP/HC) at the University of São Paulo and was approved by the Research Ethics Committee in the Analysis of Research Projects at the Hospital das Clínicas, School of Medicine, University of São Paulo, under Protocol Number 575/09. A written consent form with detailed information on the aim and protocols of the study was also approved by the same ethics committee. All parents provided written informed consent on behalf of the children involved in the study.

### MATERIALS

#### Apparatus

The experiment took place in an isolated room in the Speech-Language Pathology Clinic. Auditory tests were administered binaurally in a sound-treated booth at a level of 40 dB NS using an audiometer, headphones, and compact disks. Attention and short-term memory tests were administered using the E-Prime Professional Software to display the stimuli and collect the data. The language tasks were recorded using a JVC® Everio video camera and a Zoom H2 digital recorder for audio. Auditory intervention was delivered individually using a laptop, headphones, and specific software. The stimuli were presented binaurally at a comfortable listening level, which corresponded to a sound level of 70 dB (A). In the phonological intervention approach, children were positioned face-to-face with the speech and language pathologist to provide visual support of the therapist’s mouth. Target sounds were presented at approximately 50–60 dB HL at a distance of 1 m.

#### Outcome measures

The intervention outcomes were categorized as “auditory-sensory measures” (i.e., auditory temporal processing skills) and “cognitive measures” (i.e., attention, short-term memory, speech production, and phonological awareness skills).

***Auditory-sensory measures*.**

Frequency Pattern Test (FPT; Musiek, 1994). The FPT consists of 20 trials with ∼6-s intervals between each trial pair. Each trial consisted of three stimuli for 150 ms with an inter-stimulus interval of 200 ms. The low stimulus (L) was 880 Hz, and the high stimulus (H) was 1122 Hz. There were six possible stimulus combinations: HHL, HLL, HLH, LHL, LLH, and LHH. The children were instructed to carefully listen to all three stimuli and respond by naming them in the order in which they were presented (e.g., “low, low, high”; “high, low, low”; etc.). After the study, we calculated the percentage of correct answers. This test was administered binaurally in a sound-treated booth at a level of 40 dB NS. In non-impaired Brazilian children (ages 7–11 years-old), the expected result varies between 47.5 and 69.4% (Schochat et al., 2000).

Gap in Noise Test (GIN – Musiek et al., 2005). The GIN Test consists of stimuli with ten different gap lengths of 2–30 ms. In this test, the participants listened to segments of broadband noise that contained 0, 1, 2, or 3 silent intervals (i.e., gaps). As Musiek et al. (2005) described, the broadband noise was turned off and on instantaneously to produce gaps. Listeners were instructed to raise their hands each time they heard a gap. Gaps were separated by at least 500 ms for each trial. The test was performed in a sound-treated booth at a level of 40 dB NS. The task consisted of 35 trials presented binaurally. In non-impaired Brazilian children (ages 8–10 years-old), the expected result is ∼6.1 ms (Amaral and Colella-Santos, 2010).

***Cognitive measures*.**

Auditory and Visual Attention Tests (Murphy et al., 2014). In both tests, performance is assessed using tasks that require participants to remain prepared to respond to infrequent targets (e.g., digits, letters, or symbols) over an extended period of time. In the present research, both tests were developed using E-Prime Professional software. In the visual test, digits between 1 and 7 were presented on a screen and participants pressed a button as quickly as possible each time a 1 or 5 appeared. The auditory task was identical to the visual task except that the participants heard the digit spoken over a set of calibrated headphones. The stimuli were presented binaurally at a comfortable listening level corresponding to a sound pressure level of 70 dB (A). The duration of each test was ∼6 min and consisted of 210 trials. Three performance measures were compared across blocks: correct detection (HIT), false alarms (FAs: errors of omission and commission), and reaction time (RT). Participants were tested individually in a quiet, well-lit laboratory on campus. The testing session was composed of two parts: evaluation of auditory sustained attention and evaluation of visual sustained attention. The order was counterbalanced among participants. Before each section, the participants were given appropriate instructions and asked to perform approximately 15 practice trials.

Visual digit span (forward recall; Murphy et al., 2014). This task was developed using E-Prime Professional software. The digit span task begins with a series of three digits, with 12 attempts for each series. Children verbally repeat each numerical sequence after viewing the numbers on a computer screen. If the children are correct more than 50% of the time, longer series are gradually presented. The span result is the last series for which the subject’s responses were more than 50% correct.

Speech production. Assessed by the picture-naming and the word imitation tasks (Wertzner, 2004), derived from the Infantile Language Test-ABFW (Andrade et al., 2004). The picture-naming task was composed of 34 pictures of objects (24 dissyllable and 10 trisyllable words) with 90 consonants and the word imitation task was composed of 39 words (25 dissyllable and 14 trisyllable words) with 107 consonants. Two researchers transcribed each trial to ensure the accuracy of the data. There was ≥90% inter-reliability. The percentage of consonants correct – revised (PCC-R; Shriberg et al., 1997) was calculated separately for both speech production tasks by dividing the number of correct productions by the total number of consonants in the sample and multiplying by 100 to determine the production acuity of each subject.

Phonological awareness. Assessed by the *LindamoodAuditory Conceptualization Test* (LAC; Lindamood and Lindamood, 1979), adapted to the Brazilian Portuguese language (Rosal, 2002; Wertzner et al., 2014). The LAC test assesses phonological awareness skills without requiring verbal responses (children use colored blocks to represent their responses). This method provides superior information on phonological representations, as they prevent speech production errors from affecting the respondent’s performance. The test comprised two categories: phonological awareness 1 (PA1) and phonological awareness 2 (PA2). PA1 assesses perception skills through the auditory selection of speech sounds. It comprises six complex sameness/difference sequences covering three possible variations in sequence of three gross and three fine contrasts. The subject must discriminate how many sounds he or she heard in a pattern, and in what sequential order their sameness or difference occurs. Examples of this category are the sound patterns (/b/ /b/ /z/) and (/k/ /t/ /k/). PA2 assesses comprehension skills associated with the child’s ability to perceive and compare the number and order of sounds in a spoken pattern (including 12 stimuli that assess the manipulation of one phonemic change such as addition, substitution, omission, transfer, and repetition).

#### Intervention program

Because the impact of both approaches will be investigated for the group as a whole (not individually), we chose to adopt, for both interventions, more general training tasks instead of specialized training focused on specific speech difficulties or impaired auditory skills.

### AUDITORY INTERVENTION

The training focused on different auditory-sensory aspects, such as frequency discrimination, ordering, and backward masking. Each of the three tasks took ∼15 min to complete, resulting in 45 min of total training per session. The following software was used for the training tasks:

(1) Backward masking and frequency discrimination: the System for Testing Auditory Responses/STAR (Moore et al., 2008). This software was responsible for training backward masking and frequency discrimination skills. A laptop computer with headphones was used to present the stimuli. The stimuli were presented binaurally at a comfortable intensity. A three-interval, three-alternative, forced-choice oddball design was used for both tasks. In the frequency discrimination task, three sound-emitting characters were presented, one of which emitted a sound at a different frequency from the others. The objective of the task was to detect the different frequency by clicking on the corresponding character. During this activity, the degree of difficulty was automatically modified by decreasing the difference between the standard stimuli and the target through an adaptive staircase assessment. The backward masking task was performed in a similar manner. Three sound-emitting characters were presented, of which one emitted a 20-ms pulse tone target 50 ms before the noise. The goal of the task was to recognize which character emitted the pulse tone and the noise. The degree of difficulty was modified via the automatic reduction of the pulse tone intensity.
(2) Frequency ordering: sweep frequency was conducted using Auditory temporal training with non-verbal and verbal extended speech® software. This task trains both frequency discrimination and ordering skills. During the task, participants listened to two or three stimuli (depending on the task phase) and matched the stimuli to a sign on the screen. The following acoustic characteristics were presented: stimulus durations of 40–200 ms and frequencies that varied by 6.8 octaves per second. The initial and final frequencies were 0.5, 1 or 2 kHz, with an inter-stimulus interval that varied between 20 and 500 ms. The task consisted of 18 stages of varying difficulty levels (i.e., variations in the inter-stimulus interval and stimulus duration).

### PHONOLOGICAL INTERVENTION

As mentioned previously, because the impact of this approach was investigated for the group as a whole (not individually), for the present study, we designed a phonological stimulation program (PSP) for the stimulation of different sounds of the phonetic inventory. The PSP was formulated to expose the participants to all sounds from the Brazilian Portuguese system independent of the phonological processes observed during evaluations such that phonological acquisition could occur gradually over a short period of time (12 sessions of stimulation). Compared to more traditional phonological intervention approaches, the current approach is more closely linked to the Cycles Phonological Remediation Approach (Hodson and Paden, 1983, 1991), which also predicts that phonological acquisition in children with phonological disorders is gradual, as in typically developing children, and should be associated with kinesthetic and auditory sensations in order to acquire new patterns. Therefore, this approach intends to increase the child’s intelligibility by facilitating the emergence of primary target patterns for beginning cycles such as final consonants, clusters, velars, and liquids.

During the 12-weeks period of the intervention, all 21 consonantal sounds (CVs) and 13 clusters (CVC) of Brazilian Portuguese were stimulated through activities involving the auditory perception of the target sound, articulatory production, phonological organization, and metalinguistic abilities. Every 2 weeks, each child was exposed to a new specific sound pattern within CV syllables, such as stops, fricatives, liquids and nasals, as well as more complex syllables such as CVC and CCV, regardless of the child’s performance and the phonological processes observed in evaluations.

The sound patterns stimulated were as follows: sessions 1 and 2 – fricatives (/f/, /v/, /s/, /z/, /∫ /, /Ʒ/) sessions 3 and 4 – stops (/p/, /b/, /t/, /d/, / k/, /g/); sessions 5 and 6 – liquids (/l/, /Ր/, /λ/) and the velar fricative (/x/); sessions 7 and 8 – (/m/, /n/, /ɲ;/) and (/s/, /Ր/) in CVC syllables; sessions 9 and 10 – /l/ in CCV syllables and sessions 11 and 12 – /Ր/ in CCV syllables. We based the target sequence of stimuli on different studies with Brazilian Portuguese-speaking children (Wertzner, 2004; Wertzner et al., 2006, 2007), which indicate that difficulties with the liquids production followed by devoicing of fricatives and stops are the most common speech deficits in children with SSD. As the liquid sounds are complex sounds due to both its production and its occurrence in Brazilian Portuguese distribution, we chose to begin the PSP with the presentation of the fricatives followed by the stops so we could also be able to present the differentiation of the contrast between voiced and voiceless sounds. After these sounds, we presented the liquids and the velar fricative followed by the most complex syllables (CVC and CCV) to finish the program.

A variety of tasks were used during the PSP, some of which will be highlighted here. One of the auditory perception tasks was to read three words beginning with each target sound to the child and then perform auditory recognition training for the sounds. In the articulatory tasks, the child had to pay attention to the sound and how the sound was produced by the researcher. Explanations regarding the sound’s production were also given. Then, the child had to name specific objects beginning with the target sounds. In the tasks concerning phonological organization, the researcher asked the child to create a sentence including the name of a picture. Metaphonological tasks including syllable, rhyme, and alliteration activities were also performed in addition to phonological memory tasks with words beginning with the target sounds.

### METHODS

#### Participants

A total of 19 children diagnosed with SSD were invited to participate in this study. The children were recruited through the Laboratory of Investigation in Phonology within the Department of Physical Therapy, Speech-Language Pathology, Audiology and Occupational Therapy at the School of Medicine at the University of São Paulo. The children were diagnosed using the phonology test (Wertzner, 2004) derived from the Infantile Language Test-ABFW (Andrade et al., 2004). Diagnosis of a SSD was made by the by the presence of phonological impairments, which were determined by the presence of phonological processes that were not age expected and the absence of impairment in the other language areas (vocabulary, pragmatics, and fluency), which are also measured using the Infantile Language Test-ABFW (Andrade et al., 2004). After diagnosis, the PCC-R (Shriberg et al., 1997) was determined based on the speech samples obtained by picture-naming and an imitation of word tasks from the phonology test (Wertzner, 2004). This quantitative measure was chosen because it is highly sensitive to differences in phonological deficits and provides information pertaining to the two primary error types: omissions and substitutions (Shriberg et al., 1997). The children were monolingual Brazilian-Portuguese speakers and were not undergoing rehabilitation.

The inclusion criteria were as follows: age between 7 and 12 years, diagnoses of a SSD using the phonological output/speech production test described above; no deficits in other language areas (vocabulary, pragmatics, and fluency), IQ > 80 (based on the WISC-IV); and no familial or personal history of diagnosed or suspected auditory, otological or neurological disorders or injuries. This specific age range was chosen because the complexity of the some auditory tasks included in the auditory intervention, which would not necessarily be easily comprehended by younger children. In addition, participants were required to demonstrate normal tympanometry and acoustic reflexes. Auditory sensitivity was required to be within normal limits (≤15 dB HL for octave frequencies from 250 to 8000 Hz) and symmetrical (interaural differences ≤5 dB HL at each frequency). In order to investigate these inclusion criteria, they were required to pass a series of inclusion tests consisting of a parent questionnaire, an audiological evaluation, language tests and a non-verbal IQ test (the Raven test of Colored Progressive Matrices with Brazilian norms (Angelini et al., 1999) and a conversion table of IQ values (Strauss et al., 2006).

The results of these tests (i.e., the IQ test and audiological evaluation) led to the exclusion of two children. Then, the selected children were randomly assigned into either the auditory intervention group (AG, *n* = 10) or the phonological intervention group (PG, *n* = 7). **Table 1** displays the characteristics of these two groups (gender, age, IQ, and language skills).

**Table 1:** Performance characteristics of the AG and PG on the screening battery.

Variables	AG	(*n* = 10)	PG (*n* = 7)	*p*
**Gender (*n*)**
Girls	2	3	
Boys	8	4	
**Age (M ± SD)**	7.7 ± 1.2	8.8 ± 1.06	0.053
**Speech production tasks (M ± SD)**
Picture-naming (%)	77.3 ± 12.1	87.9 ± 7.89	0.06
Imitation of words (%)	76.7 ± 10.9	90.5 ± 8.40	0.01*
**Short-term memory (M ± SD)**
Digit Span	3.9 ± 0.7	4.4 ± 0.78	0.17
**Auditory tests (M ± SD)**
Audiological evaluation	No alteration	No alteration	
Frequency Pattern Test	43 ± 18	54.3 ± 17.4	0.21
Gap in Noise Test	9 ± 6	8.3 ± 5.5	0.80
**IQ score (Raven test)**	108.2 ± 8.7	104.5 ± 7.4	0.35

Speech production tasks: percent consonants correct for both picture- naming and imitation of words. AG, auditory group; PG, phonological group; M, mean; SD, standard deviation; IQ, intellectual quotient; *significant.

There were no significant inter-group differences with regard to age (*p* = 0.053), IQ (*p =* 0.35), short-term memory (*p =* 0.17), auditory processing (Frequency Pattern Test: *p =* 0.21, Gaps in Noise test: *p =* 0.80), and one of the language skills (picture-naming: *p =* 0.06). Differences were found only for imitation of words (*p =* 0.013). The significance threshold was set at *p* < 0.05 (**Table 1**).

#### Procedures

**FIGURE 1:**
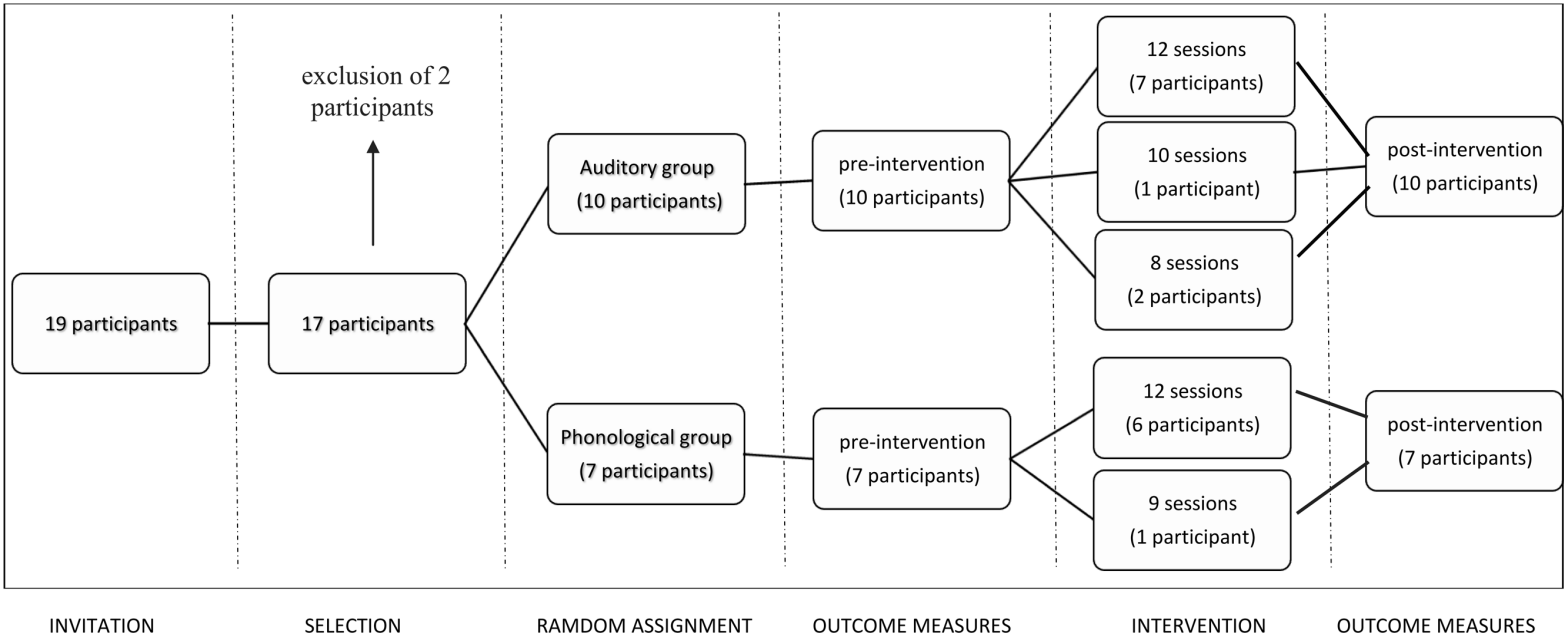
Procedures

After the groups were established, a series of tests concerning attention, short-term memory, language, and auditory processing were applied before and after the interventions (outcome measures). The characteristics regarding each of these tests are described in the Materials section. Each participant was allocated to one of the two intervention groups. Both of these approaches consisted of 12 45-min sessions twice per week, for a total of 9 h of training. The details regarding each program are also described in the Materials section. Both groups received approximately the same number of training sessions (AG: mean = 11 sessions, PG: mean = 11.4 sessions; *p =* 0.62). **Figure 1** demonstrates the sequence of procedures adopted from the initial invitation to participants until the number of completed training sessions for each group.

### STATISTICAL ANALYSIS

The data were analyzed using Minitab Statistical Software version 16.1. Non-parametric statistics were used because both groups violated the assumption of normal distribution necessary for parametric analysis. Intra- and inter-group analyses were used not only to investigate the effect of each intervention approach separately (intra-group analysis) but also to compare the level of improvements following interventions in both groups (inter-group analysis).

For the first analysis, the pre- and post-intervention performances were compared separately for each group in each of the tests (intra-group analysis using the Wilcoxon test). In the second analysis, the differences between the pre- and post-intervention performances (“improvement-following training”) were compared between both groups in each of the tests (inter-group analysis using the Mann–Whitney test). The significance threshold was set at *p* < 0.05.

## RESULTS

### INTRA-GROUP ANALYSIS

**Table 2** displays the performances in auditory-sensory and cognitive measures for both groups (pre- and post-training).

**Table 2:** Comparison pre and post-intervention period (Intra-group analysis).

Tasks	AG			PG		
	pre	post	*p*	pre	post	*p*
	M ± SD	M ± SD		M ± SD	M ± SD	
**Auditory**
*FPT (%)*	43 ± 18	64 ± 19.1	0.01*	54.3 ± 17.4	52.85 ± 14	0.95
*GIN*	9 ± 6	8.4 ± 6.3	0.05*	8.2 ± 5.5	8.4 ± 5.4	0.85
**Attention**
*Visual HIT*	56.3 ± 2.5	57.3 ± 1.3	0.31	56.6 ± 2.5	56.1 ± 3.62	0.78
*Visual FA*	2.9 ± 3.3	1.7 ± 1.1	0.28	2.4 ± 2.7	3.14 ± 1.95	0.78
*Visual RT*	716.7 ± 88.7	670.9 ± 79.1	0.03*	672,8 ± 36.7	703.53 ± 59.40	0.27
*Auditory HIT*	44.5 ± 8.3	49.2 ± 4.0	0.13	50.7 ± 4	51.42 ± 6	0.55
*Auditory FA*	10.7 ± 7.2	5.4 ± 3.7	0.03*	6.0 ± 3	3.57 ± 1.7	0.07
*Auditory RT*	1067 ± 81.4	1038 ± 61.8	0.18	1096 ± 27.3	1065 ± 48.57	0.35
**Short-term memory**
*Digit span*	3.9 ± 0.7	4.4 ± 0.7	0.05*	4.4 ± 0.7	4.5 ± 0.53	0.78
**Phonological skills**
*Picture-naming (PCC)*	77.3 ± 12.1	77.86 ± 11.4	0.72	87.9 ± 7.8	90.01 ± 9.15	0.13
*Imitation of words (PCC)*	76.7 ± 10.9	80 ± 9.8	0.10	90.5 ± 8.4	90.25 ± 8.93	0.83
*PA1-discrimination*	4.0 ± 1.6	4.6 ± 1.1	0.20	5.4 ± 0.5	5.7 ± 0.48	0.46
*PA2-manipulation*	4.4 ± 3.2	6.2 ± 3.5	0.27	8.1 ± 3.9	8.9 ± 2.1	0.50

FPT, Frequency Pattern Test; GIN, Gap in Noise; FA, false alarm; RT, reaction time, AG, auditory group; PA, phonological awareness; PCC, percentage of consonants correct; PG, phonological group; M, mean; SD, standard deviation; *significant.

#### Auditory group

The Wilcoxon test demonstrated significant differences between the pre- and post-intervention performances for both auditory measures (FPT: *p =* 0.01 and GIN: *p* = 0.05), one of the visual attention measures (RT: *p* = 0.03), one of the auditory attention measures (FA: *p* = 0.03) and digit span (*p* = 0.05). No significant differences were observed for the other outcomes (picture-naming: *p* = 0.72; imitation of words: *p* = 0.10; Visual HIT: *p* = 0.31; Visual FA: *p* = 0.28; Auditory HIT: *p* = 0.13; Auditory RT: *p* = 0.18; IB: *p* = 0.20; II: *p* = 0.27).

#### Phonological group

The Wilcoxon test demonstrated no significant differences between the pre- and post-intervention performances in any of the measures [auditory (FPT: *p* = 0.95; GIN: *p* = 0.85), short-term memory (*p* = 0.78), visual attention (HIT: *p* = 0.78; FA: *p* = 0.78; RT: *p* = 0.27), auditory attention (HIT: *p* = 0.55; FA: *p =* 0.07; RT: *p =* 0.35) and language (picture-naming: *p* = 0.13; imitation of words: *p =* 0.83; IB: *p* = 0.46; II: *p* = 0.68)].

### INTER-GROUP ANALYSIS

With regard to the auditory-sensory measures, the Mann–Whitney test showed a significant difference between the gains in both groups for both auditory measures (PF: *p* = 0.01; GIN: *p* = 0.02).

With regard to the cognitive measures, the Mann–Whitney test demonstrated significant differences between the gains in both groups for visual RT (*p* = 0.02) and no significant differences between gains in both groups for language tasks (IB: *p* = 0.58; II: *p* = 0.52; picture-naming task: *p* = 0.69; imitation of words task: *p* = 0.32), the short-term memory task (*p* = 0.45) and the other auditory and visual attention measures (visual HIT: *p* = 0.72; visual FA: *p =* 0.41; auditory HIT: *p* = 0.35; auditory FA: *p* = 0.88; auditory RT: *p* = 1.0).

**FIGURE 2:**
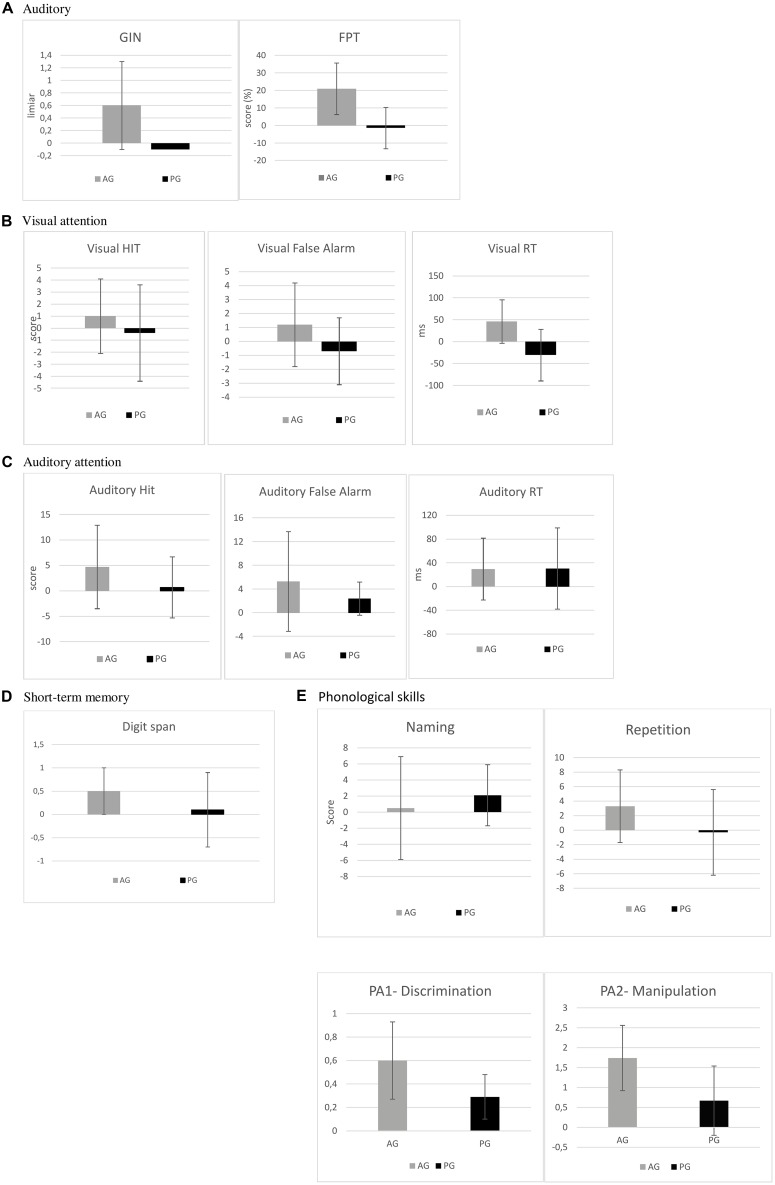
**Gains in score from pre to post-testing for the AG and PG group. (A)** Percentage of increase from pre to post-testing in FPT and decrease of GIN threshold from pre to post-testing. **(B,C)** Increase of score in Visual and Auditory HIT, decrease of auditory and visual FA, decrease of auditory and visual RT (ms), **(D)** increase of digit span, **(E)** percentage of increase in picture-naming, imitation, IB and II.

To summarize, intra-group analysis demonstrated that the auditory intervention group showed significant gains in both auditory and cognitive measures, whereas no significant gain was observed in the phonological intervention group. Inter-group analysis demonstrated significant differences between the improvement following training for both groups, with a more pronounced gain for the non-linguistic auditory temporal intervention in one of the visual attention measures and both auditory measures. No significant improvement on phonological skills was observed in both analysis in any of the groups (**Table 3** and **Figure 2**).

**Table 3:** Comparison between gains in both groups (Inter-group analysis).

	Gain			
Tasks	AG	PG	*p*
	M ± SD	M ± SD	
**Auditory**
*FPT (%)*	21 ± 14.7	–1.4 ± 11.8	0.01*
*GIN*	0.6 ± 0.7	–0.2 ± 0.1	0.02*
**Attention**
*Visual HIT*	1 ± 3.1	–0.4 ± 4	0.72
*Visual FA*	1.2 ± 3	–0.7 ± 2.4	0.41
*Visual RT*	45.8 ± 49.6	–30.7 ± 58.9	0.02*
*Auditory HIT*	4.7 ± 8.2	0.7 ± 6	0.35
*Auditory FA*	5.3 ± 8.4	2.4 ± 2.8	0.88
*Auditory RT*	29.5 ± 52	30.5 ± 68.5	1.0
**Short-term memory**
*Digit span*	0.5 ± 0.5	0.1 ± 0.8	0.45
**Phonological skills**
*Picture-naming (PCC)*	0.5 ± 6.4	2.1 ± 3.8	0.69
*Imitation of words (PCC)*	3.3 ± 5	–0.3 ± 5.9	0.32
*PA1-discrimination*	0.6 ± 1.5	0.29 ± 0.7	0.58
*PA2-manipulation*	1.74 ± 3.8	0.67 ± 3.4	0.52

FPT, Frequency Pattern Test; GIN, Gap in Noise; FA, false alarm; RT, reaction time; PA, phonological awareness; PCC, percentage of consonants correct; AG, auditory group; PG, phonological group; M, mean; SD, standard deviation; *significant.

## DISCUSSION

The purpose of this study was to compare the impact of a non-linguistic auditory and a phonological intervention approach on the phonological skills of children with SSD. Before discussing the present results, it is important to discuss the characteristics of the groups, specifically the age and the pre-training performance in phonological tasks. Although no significant differences were observed with regard to age, there was a difference of ∼1 year between the groups (children in the phonological intervention group having the highest mean age). Although several studies have corroborated the hypothesis regarding the existence of a critical period for learning (Knudsen, 2004), a difference of 1 year is insufficient to influence significant differences in the way that the learning process occurs, especially comparing 7- and 8-years-old. Murphy and Schochat (2011), for instance, observed a significant difference between the gains following auditory training only between a younger group (ages 7–10) and an older group (ages 11–14). However, the age difference in our study possibly influenced the performance on the phonological and short-term memory tasks pre intervention. This result is expected given that, even in children with SSD, these two skills improve with development (to some extent). Therefore, specifically for the imitation of words task, the phonological group had a significantly better performance than the auditory group; however, the difference between the groups in the short-term memory task was not significant. The implications of the performance of the phonological group on the phonological tests will be discussed further, with the comments concerning the improvement following training on the same tests. Regarding gender, both groups contained a higher number of boys, which corroborates previous research on the higher prevalence of SSD in boys (Shriberg et al., 1986, 1994; Wertzner and Oliveira, 2002).

The Intra-group analysis demonstrated that although no significant improvement following training was observed for the phonological group, the auditory group showed significant gains in both auditory, one of the visual and one of the auditory attention measures as well as in the digit span measures.

Regarding the auditory group, the improvements for both the FPT and GIN test were expected because the trained task in the auditory intervention approach is similar to both of these outcome measures. Thus, this improvement is likely to represent mid-transfer, that is, the learning generalization from the trained task to a different task in the same domain. Other studies, like the present research, have also demonstrated improvements following a non-linguistic auditory intervention approach in a similar trained task (Kujala et al., 2001; Murphy and Schochat, 2011). Kujala et al. (2001), for instance, used non-linguistic audiovisual computer training, with sound elements varying in pitch, duration, and intensity, in reading-impaired children. After training, improvements in a behavioral auditory frequency discrimination task were demonstrated, corroborating the results of the present research. Murphy and Schochat (2011) applied frequency discrimination training in children with dyslexia. After training, there was a significant improvement in the trained group on a similar trained task.

Despite the improvement of the auditory group on both auditory-sensory measures, no significant improvement was observed for language tasks, suggesting no generalization from non-linguistic auditory tasks to higher phonological skills. Previous research has demonstrated that this is a controversial topic. Some studies have observed improvements in verbal skills after auditory training (Kujala et al., 2001; Lakshminarayanan and Tallal, 2007; Murphy and Schochat, 2011), whereas others failed to show the same results (Halliday et al., 2012). Kujala et al. (2001), for instance, implemented an audiovisual training program including only non-linguistic stimuli for a group of 7-years-old dyslexic children (*n* = 24). The results showed that whereas before training, there were no differences in performances on reading tests between the “trained” and “untrained” groups (both composed of dyslexic children), after training, the “trained” group had better results than the “untrained” group. Electrophysiological auditory tests also showed similar results – larger amplitudes of the mismatch negativity wave were seen after training. The researchers suggested that non-linguistic auditory training, such as in the current research, can improve reading skills. In contrast, in a study conducted by Halliday et al. (2012), no learning generalization across different tasks or stimuli was found when different types of sensory training were given (auditory frequency discrimination, auditory phonetic discrimination, and visual frequency discrimination tasks). The authors concluded that learning following auditory training was specific to the task or stimulus. Most likely, these controversial results are due to the methodological differences among studies, such as the training delivered (amount of training, type of task, and stimulus), the outcome measures (how far from the trained task the effect extends) and the population (typically developing children or those with language disorders). Regarding the length and intensity of the training, for instance, we administered both training approaches over 12 sessions of 45 min each (one per week, totaling 12 weeks), whereas Kujala et al. (2001) administered 14 sessions of 10 min (twice per week, totaling 7 weeks) and Halliday et al. (2012) administered 12 sessions of 30 min (three times per week, in total 4 weeks). Although Halliday et al. (2012) provided the most intensive training, no generalization was observed from the auditory stimulus or task to higher level measure of language ability. One possible explanation was demonstrated by Molloy et al. (2012), who claimed that optimal training regimens should have short sessions spaced by several days in early learning, as done by Kujala et al. (2001), which was the only study that demonstrated learning transfer from the non-linguistic stimuli to language skills.

Despite the lack of generalization from the trained tasks to language skills, intra-group analysis demonstrated improvements in short-term memory and attention outcome measures. This result suggests a positive benefit of training on the attention and memory skills of children with SSDs; moreover, it demonstrates the influence of an auditory-sensory intervention on top–down skills. As in the present research, previous studies also reported enhanced attention skills following auditory-sensory training in different populations (Stevens et al., 2008; Soveri et al., 2013). Stevens et al. (2008) demonstrated better selective auditory attention performance following *Fast ForWord* (FFW) training in children with SLI, suggesting that the neural mechanisms of selective attention are remediated through training. Soveri et al. (2013) also demonstrated improved auditory attention in healthy adults, suggesting that auditory training can modulate the allocation of auditory attention in the adult population. It is also important to note that in the current research, the improvement in short-term memory seemed to be insufficient for the enhancement of phonological skills. This transfer may occur given that poor phonological representations of speech sound systems are often attributed to deficits that involve memory skills (Bird and Bishop, 1992; Raitano et al., 2004; Kenney et al., 2006). Because short-term memory improvements were observed only for the intra-group analysis, additional studies are necessary to better investigate this result.

Contrary to the auditory group, the phonological group exhibited no improvement, after training, in auditory-sensory measures. This result was expected given that the tasks included in the phonological intervention approach did not have a close or even underlying relationship with these auditory-sensory measures. However, the lack of improvement in phonological tasks was not expected because the phonological training tasks were similar to the phonological outcome measures; therefore, it would be reasonable to expect a more pronounced gain for the phonological group. It is possible that this result is associated with the type of phonological intervention approach adopted in this study. As noted above, the phonological intervention approach consisted of more general tasks, with no focus on the individual’s performance before the intervention (deviant or missing phonemes). Therefore, the improvement in phonological outcome measures had to be linked to learning transfer from this general stimulation to some specific deviant or missing phonological process. Previous studies have demonstrated this generalization when the phonological intervention approach was based on the child’s target speech production goals. Lousada et al. (2012), for instance, described the presence of learning generalization in a study evaluating the effectiveness of a phonological intervention approach and an articulation intervention approach in children with SSDs. A generalization probe of the trained sound or phonological process to five non-intervention words was used. The authors demonstrated that the children in the phonological group showed greater generalization to untreated words than those who received articulation therapy.

The results of the inter-group analysis demonstrated no significant difference between both groups with regard to improvement on the phonological tests following intervention. One of the issues with this comparison is that the phonological group, compared to the auditory group, had a significant better performance on the phonological tests before training. Thus, the phonological group had less chance to develop, which could negatively impact the observation of increased improvement of the phonological group following intervention. Therefore, this might be a reason for the lack of a more pronounced gain in the phonological group. However, in the intra-group analyses, in which both groups were analyzed separately, the phonological group had no significant improvement, even for phonological awareness task that included manipulation, in which the score obtained prior to intervention was only 67.5%. Thus, at least for this task, there was no ceiling effect, which means that it would be absolutely reasonable to observe a significant improvement following intervention.

The initial hypothesis of this study was that each one of the interventions would improve the performance in the trained tasks (auditory and phonological skills), leading to the learning transfer to associated tasks (language, memory, and attention skills). As previously mentioned, significant improvement in the trained tasks were observed only in the auditory group. We hypothesize that this improvement might be related to the increased similarity between the auditory training tasks and the auditory outcome measures compared to the phonological trained tasks and the phonological tests. Therefore, further studies should investigate the effect of a more specific intervention approach that focuses on specific speech difficulties/phonological processes. Despite that, previous studies has also demonstrated the positive effect of more general remediation. The auditory program FFW (Tallal et al., 1996), for instance, is one of the examples of a successful general approach given that the program comprises varied skills such as auditory temporal, phonological awareness and reading skills and it is not focused in a singular aspect. In this case, research has demonstrated generalization from more perceptual trained aspects to language skills of children with language disorder (Merzenich et al., 1996; Gaab et al., 2007). Lousada et al. (2012) also described the presence of generalization from a trained phonological process to non-trained words.

The observed transfer from the auditory training to the attention and memory skills might be related to the different characteristics of the two interventions. Whereas the auditory training was administered via a computer with fixed audiovisual tasks demanding attention and time to answer, the phonological training was administered by a speech therapist with more flexible tasks and more time to answer. With regard to the transfer to phonological skills, because no significant enhancement was observed (even with auditory-sensory improvement), the results do not corroborate the initial hypothesis, which associates auditory temporal processing and phonological skills. Therefore, although the non-linguistic auditory intervention approach appears to be the most effective intervention approach, this was insufficient to promote the enhancement of speech production and phonological awareness skills. Further studies are necessary to ascertain the extent to which auditory-sensory is involved with the etiology of SSD and the process of learning generalization across bottom–up and top–down skills.

These results are based on preliminary data from 10 participants who received auditory training and seven who received phonological training. It is clear that additional data are needed to confirm and extend these findings. Further research is also required to investigate the presence of a test-retest effect through the inclusion of a control group (non-trained group).

## Conflict of Interest Statement

The authors declare that the research was conducted in the absence of any commercial or financial relationships that could be construed as a potential conflict of interest.
